# Antimicrobial Activity of Clinically Isolated Bacterial Species Against *Staphylococcus aureus*

**DOI:** 10.3389/fmicb.2019.02977

**Published:** 2020-01-15

**Authors:** Britney L. Hardy, Garima Bansal, Katharine H. Hewlett, Arshia Arora, Scott D. Schaffer, Edwin Kamau, Jason W. Bennett, D. Scott Merrell

**Affiliations:** ^1^Department of Microbiology and Immunology, F. Edward Hébert School of Medicine, Uniformed Services University of the Health Sciences, Bethesda, MD, United States; ^2^Department of Clinical Microbiology, Walter Reed National Military Medical Center, Bethesda, MD, United States; ^3^U.S. Military HIV Research Program, Walter Reed Army Institute of Research, Silver Spring, MD, United States; ^4^Multidrug-Resistant Organism Repository and Surveillance Network, Walter Reed Army Institute of Research, Silver Spring, MD, United States; ^5^Department of Medicine, F. Edward Hébert School of Medicine, Uniformed Services University of the Health Sciences, Bethesda, MD, United States

**Keywords:** *Staphylococcus aureus*, MRSA, polymicrobial interactions, bacterial interaction, clinical isolates

## Abstract

Bacteria often exist in polymicrobial communities where they compete for limited resources. Intrinsic to this competition is the ability of some species to inhibit or kill their competitors. This phenomenon is pervasive throughout the human body where commensal bacteria block the colonization of incoming microorganisms. In this regard, molecular epidemiological and microbiota-based studies suggest that species-specific interactions play a critical role in the prevention of nasal colonization of the opportunistic pathogen *Staphylococcus aureus*. Despite this, *S. aureus* exists as part of the microbiota of ∼25% of the population, suggesting that the interplay between *S. aureus* and commensals can be complex. Microbiota studies indicate that several bacterial genera are negatively correlated with *S. aureus* colonization. While these studies paint a broad overview of bacterial presence, they often fail to identify individual species-specific interactions; a greater insight in this area could aid the development of novel antimicrobials. As a proof of concept study designed to identify individual bacterial species that possess anti*-S. aureus* activity, we screened a small collection of clinical isolates from the Walter Reed National Military Medical Center for the ability to inhibit multiple *S. aureus* strains. We found that the majority of the isolates (82%) inhibited at least one *S. aureus* strain; 23% inhibited all *S. aureus* strains tested. In total, seven isolates mediated inhibitory activity that was independent of physical contact with *S. aureus*, and seven isolates mediated bactericidal activity. 16S rRNA based-sequencing revealed that the inhibitory isolates belonged to the *Acinetobacter*, *Agromyces*, *Corynebacterium*, *Microbacteria*, *Mycobacterium*, and *Staphylococcus* genera. Unexpectedly, these included seven distinct *Acinetobacter baumannii* isolates, all of which showed heterogeneous degrees of anti-*S. aureus* activity. Defined mechanistic studies on specific isolates revealed that the inhibitory activity was retained in conditioned cell free medium (CCFM) derived from the isolates. Furthermore, CCFM obtained from *S. saprophyticus* significantly decreased mortality of *S. aureus*-infected *Galleria mellonella* caterpillars. While future studies will seek to define the molecular mechanisms of the inhibitory activities, our current findings support the study of polymicrobial interactions as a strategy to understand bacterial competition and to identify novel therapeutics against *S. aureus* and other pathogens.

## Introduction

*Staphylococcus aureus* is an opportunistic pathogen that, due to its ability to quickly adapt to harsh conditions and evade the host’s immune system, can colonize virtually any niche throughout the human body. *S. aureus* causes a variety of diseases, most frequently skin and soft tissue infections, but also systemic and toxin-mediated disease (Otto, 2010). To further exacerbate matters, numerous *S. aureus* strains are resistant to multiple antibiotics, which subsequently makes treatment more difficult. Even amongst otherwise healthy individuals, the lack of appropriate treatment often leads to more severe morbidity and higher mortality rates (Lowy, 2003). Accordingly, methicillin-resistant *S. aureus* (MRSA)-mediated disease was responsible for approximately 10,000 deaths from 2005 to 2013 in the United States (Klevens et al., 2007). Furthermore, the worldwide pervasiveness of multidrug-resistant *S. aureus* strains has led the World Health Organization to designate MRSA as a “high” threat to the global population (WHO, 2017).

Despite the propensity to cause significant morbidity and mortality, *S. aureus* exists as a part of the microbiota of approximately one-quarter of the population (Sakr et al., 2018); however, colonized individuals are more likely to develop *S. aureus*-mediated disease (Kluytmans and Wertheim, 2005). In thinking about the dynamics of colonization of the host, *S. aureus* must interact and compete with the other resident flora as a means to establish itself as a part of the microbiota of a particular niche (Burian et al., 2017). This is undoubtedly a complicated process. However, even the vast amount of currently available microbiota data has not substantially increased our current understanding of the molecular mechanisms underlying the complex interactions between resident flora and incoming pathogens like *S. aureus*. It is well-established that commensal microbes play a critical role in decreasing and preventing pathogen colonization. A well-known example of this can be found with the ability of fecal transplants from healthy donors to treat patients with recurrent *Clostridium difficile* infections; restoration of the normal gastrointestinal microbiota eliminates and prevents *C. difficile* colonization (Buffie et al., 2015).

The ability of commensal bacteria to block pathogen colonization is true at other anatomical locations as well. *S. aureus* nasal colonization in particular is greatly dependent on molecular interactions with the nasal flora (Brugger et al., 2016; Sakr et al., 2018). Indeed, the nasal cavity is a high salinity and nutrient scarce niche where resident and incoming bacteria compete for limited resources and space in a type of “bacterial warfare” (Krismer et al., 2014). These interactions are often species-specific, and commensal bacterial have been found to use a variety of mechanisms to block pathogen colonization, including the production and secretion of toxic compounds that directly kill or inhibit competitors. For example, various species from within the *Streptococcus* and *Corynebacterium* genera are inversely correlated with the presence of *S. aureus* in the nasal cavity or have been found to directly antagonize *S. aureus* (Lemon et al., 2010; Bomar et al., 2016). Even other members of the *Staphylococcus* genus have been found to negatively impact *S. aureus* viability; several coagulase-negative *Staphylococcus* (CoNS) species have evolved mechanisms to inhibit *S. aureus* colonization. Specifically, some *S. epidermidis* strains secrete a serine protease that is capable of disrupting *S. aureus* biofilm formation and blocking nasal colonization (Iwase et al., 2010). *S. hominis* and *S. epidermidis* both secrete strain-specific antimicrobial peptides that have potent selective bactericidal activity against *S. aureus* (Nakatsuji et al., 2017). Moreover, lugdunin, a novel cyclic peptide antibiotic produced by *S. lugdunensis*, has bactericidal properties against several Gram-positive pathogens, including *S. aureus*, and can prevent *S. aureus* nasal colonization (Zipperer et al., 2016). It is clear that within the context of the human nose, there is a selective pressure, even amongst closely related commensal species, to block or eliminate *S. aureus*.

Despite recent advancements detailing the negative molecular interactions that occur between *S. aureus* and the resident nasal flora, little is known about *S. aureus* interactions with bacteria isolated from other anatomical locations. Given this deficit and the fact that *S. aureus* can colonize the human body virtually ubiquitously, as a proof of concept study we set out to characterize *S. aureus* interactions with clinical bacterial isolates obtained from a variety of body sites from a diverse patient population at the Walter Reed National Military Medical Center. Herein, we show that the majority (82%, 28/34) of clinical isolates possessed some degree of *in vitro* anti-*S. aureus* activity when tested against multiple strains of *S. aureus*, including MRSA. Moreover, eight clinical isolates showed anti-*S. aureus* activity against all tested strains. Several of the clinical isolates that belonged to the *Staphylococcus* and *Corynebacterium* genera mediated contact-independent inhibitory activity against *S. aureus*. Furthermore, a portion of the clinical isolates (7/28) showed bactericidal activity against *S. aureus*. Unexpectedly, *Acinetobacter baumannii* isolates represented the most commonly identified species that produced heterogenous strain-specific anti-*S. aureus* activity. Finally, analysis of conditioned cell free medium (CCFM) from several isolates revealed that inhibitory activity was often present in the CCFM. Furthermore, CCFM derived from *S. saprophyticus* was able to reduce mortality of *S. aureus-*infected *Galleria mellonella* caterpillars. These findings suggest that *S. aureus* interactions with other bacteria are far more multifaceted than previously recognized, and strongly support the study of these interactions at the molecular level as a means to reveal novel *S. aureus* molecular targets or therapeutics.

## Materials and Methods

### Strains, Culture and Bacterial Interaction Assays

All deidentified clinical isolates were obtained as a part of a memorandum of understanding (MOU) between the Uniformed Services University of the Health Sciences (USU) and the Walter Reed National Military Medical Center (WRNMMC), Department of Clinical Microbiology. The described studies represent research Not Involving Human Subjects since all isolates were obtained from discarded clinical microbiology plates that contained samples that were obtained during routine diagnostic testing and treatment of WRNMMC patients. Both USU and WRNMMC agree and acknowledge that the activities and projects pursued under the MOU complied with the applicable rules and regulations governing human subjects research within the Department of Defense; the Institutional Review Board at WRNMMC was the IRB of record for the collection of all patient samples. Strains were maintained as −80°C freezer stocks and were cultured under the following conditions unless otherwise noted: Clinical isolates were streaked from frozen glycerol stocks on Brain Heart Infusion (BHI) agar (Becton Dickinson) supplemented with 1% Tween_80_ (BHIT, Sigma-Aldrich). *S. aureus* strains were streaked from glycerol stocks on BHI agar. Each isolate was incubated overnight at 37°C. Bacterial interaction assays were performed as previously described (Yan et al., 2013; Hardy et al., 2019). Briefly, 40 mg of *S. aureus* or a clinical isolate was directly harvested from an agar plate with a sterile inoculating loop and then re-suspended in 200 μL of sterile saline solution (Fisher Chemicals). Eight microliters of the *S. aureus* cell suspension was inoculated into 15 mL of sterile BHIT agar that had been cooled to 55°C; inoculated agar was poured into a sterile petri dish, and allowed to solidify under sterile conditions. Next, 25 μL of a clinical isolate cell suspension was spotted onto the center of an agar dish (one clinical isolate per a *S. aureus-*seeded agar plate), and was allowed to dry for 40 min under sterile conditions. The resulting plates were incubated at 28°C, and the formation of a zone of clearance (ZOC) was visually assessed at 24, 72, and 120 h. Images of the ZOC were taken with an Amersham Imager 680 (General Electric). The ZOC was defined as the distance between the edge of the clinical isolate spot and the visible edge of the clearance ring. To measure ZOC length, images were analyzed using ImageJ software (NCBI). Each clinical isolate was assessed in three independent biological replicates against *S. aureus* strains 2014.N, LAC, and Mu50 (Table 1).

**TABLE 1:** Clinical isolates and *S. aureus* strains assayed.

**Strain**	**Lab strain designation**	**Origin**	**Accession #**	**Year isolated**	**Anti-*S. aureus* activity_4_**			**Contact dependent vs. Independent**	**Bactericidal vs. Bacteriostatic**	**References**
					**2014.N**	**LAC**	**Mu50**			
***Staphylococcus aureus* test strains**										
*S. aureus* LAC	DSM1485	Blood	NC_002758.2	2005	N/A			N/A	N/A	Voyich et al. (2005)
*S. aureus* 2014.N	DSM1416	Nose	N/A	2012	N/A			N/A	N/A	Hardy et al. (2019)
*S. aureus* Mu50	DSM1633	Abscess	NC_002758.2	1997	N/A			N/A	N/A	Kuroda et al. (2001)
***Acinetobacter* clinical isolates**										
*A. baumannii*-1	DSM1675	Wound	MN175920	2016	Strong	Weak	Weak	Dependent_1_	Bactericidal_1_	This Study
*A. baumannii*-2	DSM1676	Wound	MN175921	2016	Strong	Weak	None	Dependent_1_	Bacteriostatic_1_	This Study
*A. baumannii*-3	DSM1923	Wound	MN175922	2016	Strong	Weak	Weak	Dependent_1_	Bacteriostatic_1_	This Study
*A. baumannii*-4	DSM1924	Wound	MN175925	2016	None	Weak	Weak	Not Tested	Not Tested	This Study
*A. baumannii*-5	DSM1917	Blood	MN175926	2016	None	Weak	Strong	Dependent_3_	Not Tested^∗^	This Study
*A. baumannii*-6	DSM1762	Wound	MN175924	2016	None	Weak	Strong	Dependent_3_	Bactericidal_3_	This Study
*A. baumannii*-7	DSM1918	Wound	MN175923	2016	None	Weak	Weak	Not Tested	Not Tested	This Study
***Corynebacterium* clinical isolates**										
*C. amycolatum*-1	DSM1914	Nasal	MN175942	2016	Weak	None	None	Not Tested	Not Tested	This Study
*C. amycolatum*-2	DSM1567	Nasal	MN175937	2016	Weak	None	Strong	Independent_3_	Bactericidal_3_	This Study
*C. aurimucosum*-1	DSM1560	Urine	MN175936	2016	Strong	None	Weak	Independent_1_	Bacteriostatic_1_	This Study
*C. aurimucosum*-2	DSM1678	Wound	MN175938	2016	Weak	None	Strong	Independent_3_	Bacteriostatic_3_	This Study
*C. aurimucosum*-3	DSM1912	Wound	MN175945	2016	None	None	Strong	Dependent_3_	Not Tested^∗^	This Study
*C. aurimucosum*-4	DSM1913	Wound	MN175932	2016	None	None	Weak	Not Tested	Not Tested	This Study
*C. jeikeium*	DSM1915	Would	MN175945	2016	None	Weak	None	Not Tested	Not Tested	This Study
*C. striatum*-1	DSM1564	Wound	MN175927	2016	Weak	None	Weak	Not Tested	Not Tested	This Study
*C. striatum*-2	DSM1566	Blood	MN175947	2016	Strong	None	None	Independent_1_	Bacteriostatic_1_	This Study
*C. tuberculostearicum*	DSM1925	Nasal	MN175944	2016	None	None	Weak	Not Tested	Not Tested	This Study
***Microbacterium* clinical isolates**										
*M. paraoxydans*-1	DSM1919	Nasal	MN175940	2016	None	Weak	Weak	Not Tested	Not Tested	This Study
*M. paraoxydans*-2	DSM1920	Wound	MN175935	2016	None	Weak	Weak	Not Tested	Not Tested	This Study
***Staphylococcus* clinical isolates**										
*S. epidermidis*-1	DSM1679	Would	MN175939	2016	Strong	Strong	Weak	Independent_1_	Bacteriostatic_1_	This Study
*S. epidermidis*-2	DSM1759	Wound	MN175929	2016	Strong	Strong	Weak	Dependent_1_	Bactericidal_1_	This Study
*S. epidermidis*-3	DSM1760	Wound	MN175930	2016	Strong	Strong	Weak	Independent_1_	Bactericidal_1_	This Study
*S. epidermidis*-4	DSM1922	Wound	MN175931	2016	Strong	Strong	Weak	Dependent_1_	Bactericidal_1_	This Study_1_
*S. epidermidis*-5	DSM1761	Nasal	MN175933	2016	Weak	Weak	None	Not Tested	Not Tested	This Study
*S. hominis*	DSM1916	Wound	MN175934	2016	Strong	Strong	Weak	Dependent_1_	Bactericidal_1_	This Study
*S. saprophyticus*	DSM1655	Urine	MN175941	2016	Weak	Strong	Weak	Independent_2_	Bactericidal_2_	This Study
**Other clinical isolates**										
*Agromyces* sp. 3098BRRJ	DSM1921	Wound	MN175928	2016	None	None	Weak	Not Tested	Not Tested	This Study
*Mycobacterium yunnanensis*	DSM1677	Wound	MN175946	2016	Strong	None	Weak	Dependent_1_	Bacteriostatic_1_	This Study

*Subscript “1” indicates assay was tested against *S. aureus* 2014.N. Subscript “2” indicates assay was tested against *S. aureus* LAC. Subscript “3” indicates assay was tested against *S. aureus* Mu50. Subscript “4” indicates strong anti-*S. aureus* activity defined as follows: ZOC was completely transparent, ≥2 mm, and a defined edge at 72 h. Weak anti-*S. aureus* was defined as follows: ZOC was not completely transparent, with a hazy and undefined edge at 72 h. None indicates a ZOC was not present at 72 h. A “^∗^” indicates that the ZOC was too small to perform Bactericidal vs. Bacteriostatic assays. “N/A” denotes information that was unavailable or not applicable.*

### DNA Extraction, Amplification, Cloning, and 16S rRNA Gene Sequencing

All clinical isolates that possessed anti-*S. aureus* activity (28/34) were streaked from frozen glycerol stocks on BHIT agar and incubated overnight at 37°C. Single colonies of each isolate were subcultured in 2 mL of BHIT broth and incubated at 37°C with shaking for 24–48 h. Overnight broth cultures were pelleted by centrifugation and re-suspended in 0.2 mL of Phosphate Buffer Solution (PBS, Fisher Chemicals). Cell suspensions were lysed in a Bullet Blender Homogenizer for 5 min by mechanical disruption in bead-beater tubes that contained 0.1 mm sterile glass beads. Genomic DNA was extracted from lysed cells suspensions with the Wizard Genomic DNA Purification Kit (Promega) according to the manufacturer’s instructions.

Purified genomic DNA from each sample was subjected to PCR amplification of the 16S rRNA gene using the 8F (5′ AGAGTTTGATCCTGGCTCAG 3′) and 1492R (5′ GGTTACCTTGTTACGACTT 3′) primers. PCR mixtures (25 μL) contained 5X Phusion HF buffer, 200 mM of each dNTP, 0.5 μM of each primer, and 0.02 U/μL of Phusion DNA polymerase. PCR amplification was performed with the following reaction conditions: 98°C for 30 s, 30 cycles of 98°C for 5 s, 51°C for 30 s, 72°C for 1 min 30 s, with a final elongation step of 72°C for 5 min. The PCR amplified products were visualized on a 1% agarose gel to confirm the presence of an approximately 1,500 base pair band.

PCR products were purified using the QIAquick PCR purification kit according to the manufacturer’s instructions. Purified PCR products were polyadenylated utilizing the A-tailing procedure; reaction components (10 μL), including PCR-amplified DNA, 10X ThermoPol Buffer, 1mM dATP, and Taq DNA Polymerase, were incubated at 70°C for 30 min. A-tailed PCR products were subsequently cloned into the pGEM-T Easy vector according to the manufacturer’s instructions (Promega). Ligation products were transformed into *E. coli* TOP10 CaCl_2_ chemically competent cells. Transformants with the desired insert were isolated via “blue/white” selection on LB (Luria-Bertani) agar supplemented with ampicillin (100 μg/mL), X-gal (40 μg/mL) and IPTG (1 μM). To confirm the presence of the correct insert, colony PCR was performed on at least five white colonies per transformation using the GoTaq Green Master Mix (Promega) and pGEM-T Easy specific T7 (5′ GGGTTTTCCCAGTCACGA 3′) and SP6 (5′ GCACCCCAGGCTTTACAC 3′) primers with the following PCR conditions: 95°C for 3 min, 30 cycles of 95°C for 30 s, 45°C for 30 s, 72°C for 1 min 30 s, with a final elongation step of 72°C for 5 min. White colonies that contained the correct insert were cultured overnight in LB Broth plus ampicillin (100 μg/mL) with shaking. Plasmids were purified using QIAprep Spin Miniprep Kit (Qiagen) according to the manufacturer’s instructions and then used for sequencing.

As previously described (Johnson et al., 2016), to ensure near full-length coverage of the 16S rRNA gene, six individual sequencing reactions were performed on purified plasmids using the following primers: T7, SP6, 8F, 1492R, 515F (5′ GTGYCAGCMGCCGCGGTA 3′), and 806R (5′ AGAGTTTGATCCTGGCTCAG 3′). Sequence reads were manually assembled into a double stranded near full length 16S rRNA gene sequence, and taxonomic information was assigned after comparison with other 16S rRNA gene sequences in the Ribosomal Database Project (RDP)^1^ and GenBank^2^ using the Basic Local Alignment Search Tool (BLAST). The 16S rRNA gene sequences of all the strains speciated in this study were deposited in GenBank and assigned accession numbers. Strain descriptions, species identification, and accession numbers can be found in Table 1.

### Contact-Dependent Assays

Strongly inhibitory clinical isolates were defined as follows: a ZOC that was visibly transparent, at least 2 mm in length, and with a defined edge. These isolates (17/28) were assayed to determine if anti-*S. aureus* activity was dependent on direct physical contact between the bacteria; in each case, the activity of each clinical isolate was tested against the *S. aureus* strain for which the strongest ZOC was obtained in the absence of a filter disk. A sterile 0.2 μm filter disk was placed on top of the BHIT agar that had been seeded with *S. aureus*; each clinical isolate was then individually spotted on top of the filter disk so that none of the cell suspension physically touched the *S*. *aureus* seeded agar plate. Plates were incubated at 28°C and were visually assessed at 24, 72, and 120 h for the absence or presence of a ZOC. The absence of a ZOC in the presence of a filter disk indicates that physical contact is necessary for anti-*S. aureus* activity against the corresponding most sensitive *S. aureus* strain. Clinical isolates were assessed in three independent biological replicates.

### Recovery of *S. aureus* From ZOC

To determine if anti-*S. aureus* activity was bacteriostatic (growth inhibition) or bactericidal (killing), *S. aureus* survival and growth was monitored as compared to the original inoculum. Immediately after the plates solidified and before a clinical isolate was spotted, five-milligram punches of *S. aureus-*seeded agar were taken with a sterile pipette tip as a means to enumerate *S. aureus* colony forming units (CFU) present at T0. Bacterial interaction assays were performed with 15/28 strongly inhibitory clinical isolates as described above. Two isolates that produced a defined and transparent ZOC, but exactly 2 mm in length, were excluded from these experiments as the ZOC produced against *S. aureus* was too small to accurately extract agar punches. Each strongly inhibitory clinical isolate was tested against the *S. aureus* strain for which the strongest ZOC was produced. After 48 h (T48) of incubation at 28°C, five-milligram punches of agar directly adjacent to the clinical isolate spot (Inside ZOC) or at the edge of the petri dish (Outside ZOC) were again taken with a sterile pipette tip. To determine the number of *S. aureus* CFU present in an agar punch, punches were resuspended in 1 mL of BHI broth and heated to 55°C for 10 min. 10-fold serial dilutions of each suspension were prepared in PBS and then plated on Mannitol Salt Agar (MSA, Criterion). Plates were incubated at 37°C overnight, and recovered colonies were quantified. The number of CFU present in the 1 mL original suspension was calculated, and the fold change from T0 was calculated as follows: (Number of CFU present Inside or Outside ZOC at T48/Number of CFU present at T0). Fold change values less than 1 indicate bactericidal activity; *S. aureus* CFU recovered in an agar punch at T48 was less than the *S. aureus* CFU recovered in an agar punch at T0. Contact-dependent experiments were completed in three independent biological replicates.

### Conditioned Cell Free Medium (CCFM) Preparation and Disk Diffusion Assays

Clinical isolates that produced contact-independent bactericidal anti-*S. aureus* activity (*C. amy*-2, *S. sap*, and *S. epi*-3) were independently cultured in 10 mL BHIT broth overnight at 37°C with shaking at 190 rpm. Cultures were pelleted by centrifugation, and the supernatant was filter sterilized with a.2 μm filter (Corning). One-milliliter of sterile supernatant was retained, and the remaining supernatant was concentrated (50X) with ammonium sulfate precipitation as previously described (Hardy et al., 2019). For heat-treatments, 50 μL aliquots of unconcentrated or 50X CCFM were incubated at 90°C for 10 min, then allowed to cool. For the disk diffusion assays, the *S. aureus* strain that was most sensitive to the corresponding inhibitory activity (*C. amy*-2/Mu50, *S. sap*/LAC, and *S. epi*-3/LAC) was cultured on BHI agar overnight at 37°C. The following day, the plate-grown cells were recovered and diluted to 1 × 10^8^ cells/ml (OD_600_ 0.1) in BHI broth. A sterile swab was then used to spread the *S. aureus* cell suspension on BHIT agar as a lawn. The plate was allowed to dry in a laminar flow hood for 30 min. Next, a sterile 5 mm diffusion disk was placed on top of the *S. aureus* lawn, and 50 μL of unconcentrated CCFM or 50X CCFM was inoculated onto the disk. Plates were incubated at 28°C, and images were taken after 72 h of incubation. Disk diffusion assays were conducted in three independent biological replicates.

### *S. aureus* Infection and CCFM Treatment of *Galleria mellonella* Caterpillars

*Staphylococcus aureus* strains 2014.N, Mu50, and LAC were cultured overnight on BHI agar at 37°C. The following day, *S. aureus* cells were recovered and diluted to 1 × 10^8^ cells/ml (OD_600_ 0.1) in PBS. Total CFU were then further adjusted to obtain the required doses; i.e., 10^7^ CFU or 10^6^ CFU in 5 μL of PBS + 0.01% bromophenol dye. For infections, *Galleria mellonella* caterpillars (Vanderhorst Wholesale Inc) were utilized within 1 day of receipt. Caterpillars between 200 and 300 mg were chosen for infection. The injections were carried out as described previously (Desbois and Coote, 2011) with minor adaptations. Briefly, 5 μL of inoculum that contained 10^7^ or 10^6^ total CFU of *S. aureus* was injected into the last left proleg using a 10 μL glass syringe (Hamilton) fitted with a 31G needle. For caterpillars that were treated with CCFM, the caterpillars were maintained at room temperature for 1 h following the *S. aureus* injection, then refrigerated at 4°C for 12 min and then injected with 5 μL of freshly prepared 50X CCFM from *S. sap* or *S. epi*-3 (treated) or 50X concentrated BHIT (sham treated). These injections were into the last right proleg. All caterpillars were incubated at 37°C, and survival was monitored over 120 h. Untouched, and PBS injected caterpillars were included as controls. Data found in Figures 7A,B represent two completely independent biological replicates (*n* = 15 caterpillars) performed with different batches of caterpillars. Data found in Figure 7C represent a single batch of caterpillars, but two independently derived batches of CCFM (*n* = 15 caterpillars)/CCFM preparation. Kaplan–Meier survival curves were compared between groups using the Mantel–Cox test with Holm’s correction for multiple comparisons (excluding Untouched and PBS negative controls). An alpha value of 0.05 was considered statistically significant.

## Results

### Activity of Clinical Bacterial Isolates Against *S. aureus*

**FIGURE 1:**
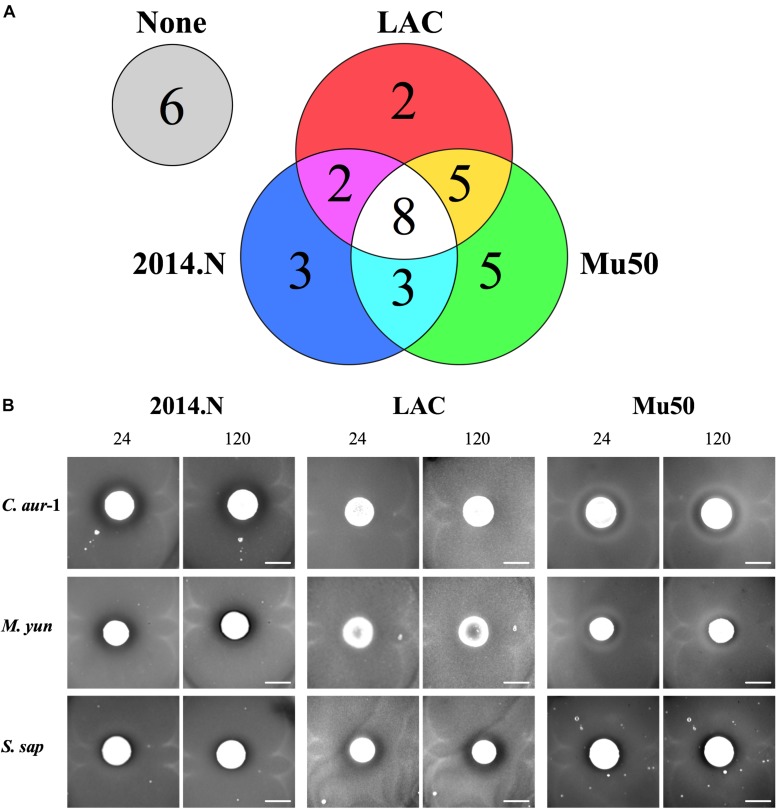
Bacterial species isolated from patients at the Walter Reed National Military Medical Center inhibit *Staphylococcus aureus* growth. **(A)** Venn Diagram of anti-*S. aureus* activity: thirty-four clinical isolates were screened for anti-*S. aureus* activity in an *in vitro* bacterial interaction assays against *S. aureus* strains 2014.N, LAC and Mu50. 28/34 isolates tested possessed anti-*S. aureus* activity against at least one *S. aureus* strain. 6/34 possessed no activity against *S. aureus*. 8/34 inhibited growth of all *S. aureus* strains tested while the remaining 20 strains showed activity against one or two of the *S. aureus* strains as indicated in the Venn Diagram. **(B)** Representative Bacterial Interaction Assays: *C. aurimucosum* (*C. aur*-1), *M. yunnanensis* (*M. yun*), or *S. saprophyticus* (*S. sap*) were spotted onto an agar plate that had been seeded with the indicated *S. aureus* strains (2014.N, LAC, or Mu50). Plates were incubated at 28°C, and images were taken at 24, 72, and 120 h (24 and 120 h images are shown). Images are representative of three independent biological replicates. Scale bar = 10 mm and is the same in the corresponding 24 h and 120 images; in some cases the 120 h spots appear larger than the 24 h spots due to growth of the bacteria within the spots.

Polymicrobial interactions within the human host are complex and dynamic. Numerous studies have shown that several genera that inhabit the skin and nasal cavity prevent the colonization of opportunistic pathogens (Jarraud et al., 2002; Bomar et al., 2016). However, these studies often focus on specific anatomical locations and do not represent the host as one environmental niche. Given this, we questioned whether bacterial species isolated from a diverse patient population and a variety of body sites would display antagonistic interactions against *S. aureus*. To this end, we obtained a collection of clinical isolates (Table 1) from the WRNMMC Clinical Microbiology Lab and assayed *in vitro* anti-*S. aureus* activity utilizing a bacterial interaction assay (Hardy et al., 2019). As prior studies have shown that antagonistic polymicrobial interactions are often strain-specific and because we previously showed that *Corynebacterium pseudodiphtheriticum*, a common skin and nasal commensal microbe, mediates heterotypic bactericidal activity against specific *S. aureus* strains (Hardy et al., 2019), we assayed anti-*S. aureus* activity against three phenotypically different *S. aureus* strains: *S. aureus* LAC (Community-Acquired, MRSA), *S. aureus* Mu50 (Hospital-Acquired, MRSA) and 2014.N (Methicillin-Sensitive *S. aureus*), a recently acquired nasal isolate (Table 1). To this end, 34 individual clinical isolates were assessed against each *S. aureus* strain in the bacterial interaction assays; appearance of a visible zone of clearance (ZOC) around the clinical isolate was considered a positive indicator of anti-*S. aureus* activity. While we found that six clinical isolates showed no anti-*S. aureus* activity, the majority (28/34, 82%) of tested clinical isolates possessed inhibitory activity against at least one of the *S. aureus* strains (Figure 1A). Furthermore, eight of the clinical isolates were able to inhibit the growth of all tested *S. aureus* strains. As expected, many of the clinical isolates mediated inhibitory activity in a *S. aureus* strain-specific manner: three clinical isolates only inhibited 2014.N, five only inhibited Mu50, and two only inhibited LAC (Figure 1A).

The species of the 28 isolates that exhibited anti-*S.aureus* activity were next identified via cloning and sequencing of the 16S rRNA gene; sequences were deposited into GenBank and accession numbers are available in Table 1. Analysis of the species information combined with the bacterial interaction assays revealed several types of ZOCs that developed over time (Figure 1B). For example, co-incubation of *Corynebacterium aurimucosum* (*C. aur*-1) or *Mycobacterium yunnanensis* (*M. yun*) with *S. aureus* 2014.N or Mu50 resulted in a diffused and moderately sized ZOC; a ZOC did not develop upon co-incubation with *S. aureus* LAC for either clinical isolate (Figure 1B). In contrast, co-incubation of *Staphylococcus saprophyticus* (*S. sap*) with *S. aureus* LAC resulted in a defined and transparent ZOC, while only a modest and hazy ZOC was produced against *S. aureus* 2014.N and Mu50.

**FIGURE 2:**
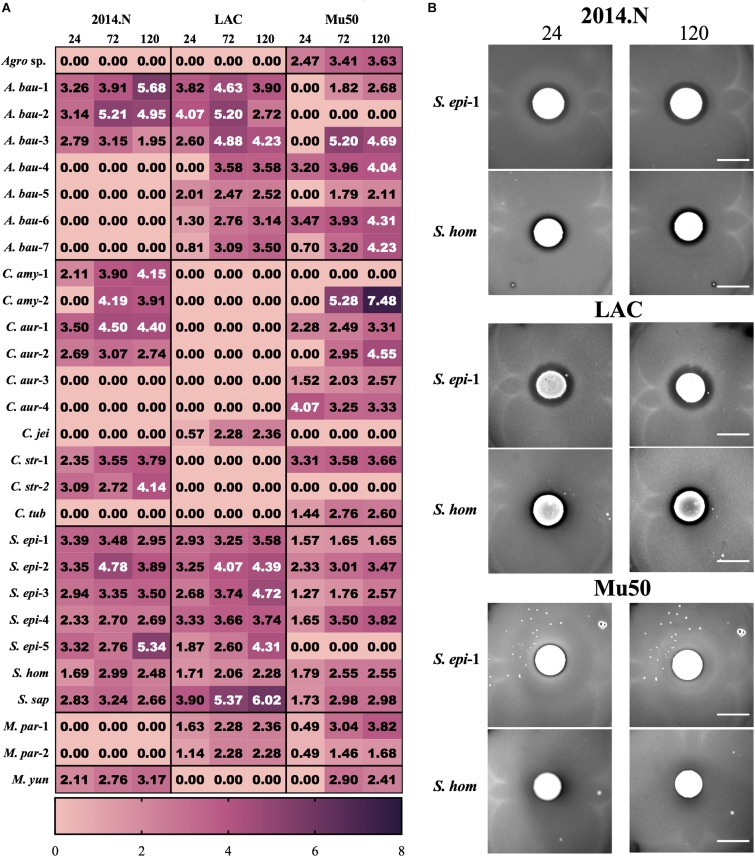
Clinical isolates mediate strain-specific anti-*S. aureus* activity. **(A)** Heat map showing the activity of the indicated clinical isolate against the indicated *S. aureus* strains: The Zone of Clearance (ZOC) was defined as the distance between the edge of the clinical isolate spot to the visible edge of the *S. aureus* ring of clearance. The ZOC was measured using ImageJ software (NCBI) and each value within a box represents the arithmetic mean (in millimeters) of three independent biological replicates measured temporally (24, 72, and 120 h). Clinical isolate species names have been abbreviated as follows: *C. aurimucosum* (*C. aur*), *C. striatum* (*C. str*), *C. amycolatum* (*C. amy*), *C. jeikeium* (*C. jei*), *C. tuberculostearicum* (*C. tub*), *S. epidermidis* (*S. epi*), *S. saprophyticus* (*S. sap*), *S. hominis* (*S. hom*), *A*. *baumannii* (*A. bau*), *M. paraoxydans* (*M. par*), *M. yunnanensis* (*M. yun*), and *Agromyces* sp. 3098BRRJ (*Agro* sp.). Additionally, a number preceded by a – is used to indicate an independent isolate of the indicated species. **(B)** Example of strain-specific activity: *S. epidermidis* (*S. epi*-1) and *S. hominis* (*S. hom*) were co-incubated with agar plates seeded with *S. aureus* strains 2014.N, LAC or Mu50. Images of the ZOC were taken after 24 and 120 h of incubation at 28°C and are representative of three independent biological replicates. Scale bar = 10 mm and is the same in the corresponding 24 h and 120 images; in some cases the 120 h spots appear larger than the 24 h spots due to growth of the bacteria within the spots.

Temporal quantification of ZOC length additionally revealed distinct patterns of interactions between each clinical isolate and each *S. aureus* strain. For the majority of the isolates, the ZOC length either remained constant or increased over time (Figure 2A). In support of the literature that suggests that some members of the *Corynebacterium* genus promote negative interactions with *S. aureus* (Yan et al., 2013; Hardy et al., 2019), numerous inhibitory isolates were speciated to be members of the *Corynebacterium* genus. These isolates tended to show anti-*S. aureus* activity selectively against strains 2014.N and Mu50; only one *Corynebacterium* isolate, *C. jeikeium* (*C. jei*), inhibited *S. aureus* LAC growth, but neither 2014.N or Mu50. Previous reports have also shown that several CoNS prevent *S. aureus* colonization by inhibiting growth or by direct killing (Iwase et al., 2010; Zipperer et al., 2016; Nakatsuji et al., 2017). In support of this, numerous Staphylococcal isolates were identified and possessed activity against *S. aureus*. These isolates generally mediated robust activity against *S. aureus* 2014.N and LAC, but only modest anti-*S. aureus* activity against Mu50 (Figures 2A,B). For example, *Staphylococcus epidermidis* (*S. epi*-1) and *S. hominis* (*S. hom*) produced defined and transparent ZOCs against 2014.N and LAC, but a comparatively small ZOC was produced against Mu50. Taken together, these results support the current hypothesis that antagonistic interactions with *S. aureus* are often strain-specific. As it would account for the differences in sensitivity amongst the various *S. aureus* strains, this may indicate that the *S. aureus* molecular target(s) of each inhibitory isolate is strain-specific and/or differentially expressed between the various *S. aureus* strains.

In addition to the expected members of the *Corynebacterium* and *Staphylococcus* genera, several clinical isolates that are not typically associated with the human microbiota were found to have anti-*S. aureus* activity. For example, there are few reports of the clinical isolation of *Microbacterium* species (Laffineur et al., 2003). However, *M. paraoxydans*, a pathogen of various fish species (Soto-Rodriguez et al., 2013), was recovered from two separate patients and both isolates possessed anti-*S. aureus* activity against strains LAC and Mu50 (Figure 2A). Similarly, *Agromyces*, a common soil microbe, also mediated anti-*S. aureus* activity against Mu50 (Figure 2A). These data indicate that antagonistic interactions with *S. aureus* are not limited to conventional members of the human microbiota that would have been under evolutionary pressure to evolve mechanisms to compete with *S. aureus*.

### Heterotypic Inhibitory Activity of *A. baumannii* Against *S. aureus*

**FIGURE 3:**
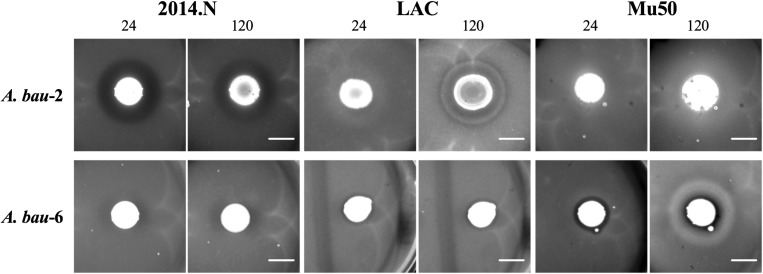
Independent *Acinetobacter baumannii* isolates produce strain-specific anti-*S. aureus* activity. Individual *A. baumannii* (*A. bau*-2 and *A. bau*-6) isolates were spotted onto an agar plate that had been seeded with various *S. aureus* strains (2014.N, LAC, or Mu50). Plates were incubated at 28°C, and images were taken at 24, 72, and 120 h (24 and 120 h images are shown). Images are representative of three independent biological replicates. Scale bar = 10 mm and is the same in the corresponding 24 h and 120 images; in some cases the 120 h spots appear larger than the 24 h spots due to growth of the bacteria within the spots.

While *Acinetobacter baumannii* and *S. aureus* have been frequently co-isolated from wounds (Furuno et al., 2008; Castellanos et al., 2019), to our knowledge there is no published evidence that *A. baumannii* possesses any inhibitory activity against *S. aureus*. Thus, we were surprised that *A. baumannii* isolates represented ∼20% (7/34) of the clinical isolates that showed anti-*S. aureus* activity (Figures 2A, 3). Though not certain, this large representation of *A. baumannii* clinical isolates may be a result of the “wounded warrior” patient population that is often treated at WRNMMC. Of the seven *A. baumannii* isolates, two (*A. bau*-1 and *A. bau*-3) possessed inhibitory activity against all tested *S. aureus* strains. The remaining five *A. baumannii* mediated anti-*S. aureus* activity against at least two strains (Figure 2A).

The type of ZOC produced by *A. baumannii* varied and was largely dependent on the *S. aureus* strain being tested. For example, *A. bau*-2 produced a large and defined ZOC against 2014.N, a large and hazy ZOC against LAC, and no ZOC against Mu50 (Figure 3). In contrast, *A. bau*-6 produced a moderately sized and very defined ZOC against Mu50, a small and hazy ZOC against LAC, and no ZOC against 2014.N. Taken together, these data indicate that *A. baumannii* possesses heterogeneous strain-specific anti-*S. aureus* activity. This may in turn indicate that *A. baumannii* utilizes multiple independently evolved mechanisms to compete with *S. aureus* or that the target(s) of anti-*S. aureus* activity are differentially expressed between *S. aureus* strains.

### Characterization of Contact-Dependent and Bactericidal Anti-*S. aureus* Activity

**FIGURE 4:**
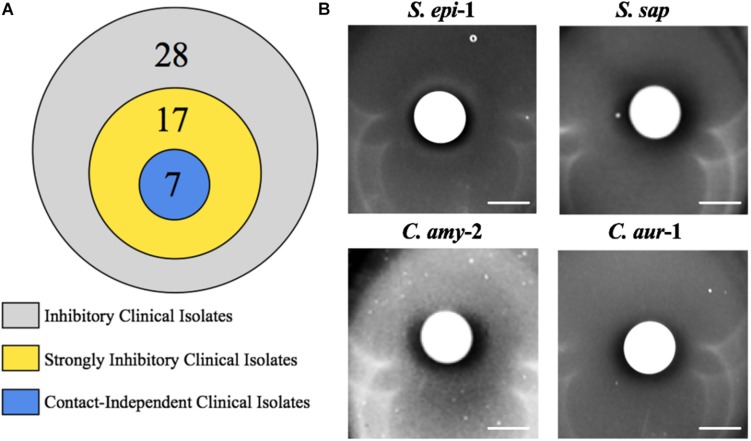
Select clinical isolates mediate contact-independent anti-*S. aureus* activity. **(A)** Strongly inhibitory clinical isolates (17/28) were defined as follows: visibly transparent ZOC of at least 2 mm with a defined edge. **(B)** A 0.2 μm filter was placed on top of BHIT agar plates seeded with *S. aureus* (*S. epi*-1 and *S. sap* were incubated with *S. aureus* LAC, *C. amy*-2 was incubated with *S. aureus* Mu50, and *C. aur*-1 was incubated with *S. aureus* 2014.N). A clinical isolate (as described above) was then spotted on top of the filter paper such that the clinical isolate and the *S. aureus* seeded agar plate were physically separated. Images of the ZOC were taken after 120 h of incubation at 28°C. Images are representative of three independent biological replicates. Scale bar = 10 mm.

Commensal bacteria utilize a wide variety of molecular mechanisms to compete with other microbes; these include both contact dependent and independent mechanisms (Brugger et al., 2016). Thus, we sought to determine whether the observed anti-*S. aureus* activity of the clinical isolates required physical bacterial interaction. Of the 28 strains that displayed activity, we focused our efforts on the 17 clinical isolates that showed strong inhibitory activity; these strains produced a defined and transparent ZOC against *S. aureus* that was greater than or equal to 2 mm. To this end, bacterial interaction assays were repeated, but the clinical isolate was separated from the *S. aureus* seeded agar with a 0.2 μm filter disk. A ZOC still formed for 41% (7/17) of the tested clinical isolates (Figure 4A), indicating that anti-*S. aureus* activity was contact-independent. The clinical isolates that mediated contact-independent anti-*S. aureus* activity were restricted to the *Staphylococcus* and *Corynebacterium* genera (Figure 4B and data not shown). For example, two independently recovered *S. epidermidis* isolates (*S. epi*-1 and *S. epi*-3) and *S. saprophyticus* (*S. sap*) mediated robust contact-independent inhibitory activity (Figure 4B and data not shown). In addition, four *Corynebacterium* species (*C. amy*-2, *C. aur*-1, *C. aur-2*, and *C. str*-1) produced moderate inhibitory activity in the presence of a filter disk (Figure 4B and data not shown). Taken together, these data indicate that the various isolates can use both contact-dependent and contact-independent mechanisms as a means to inhibit *S. aureus* growth.

**FIGURE 5:**
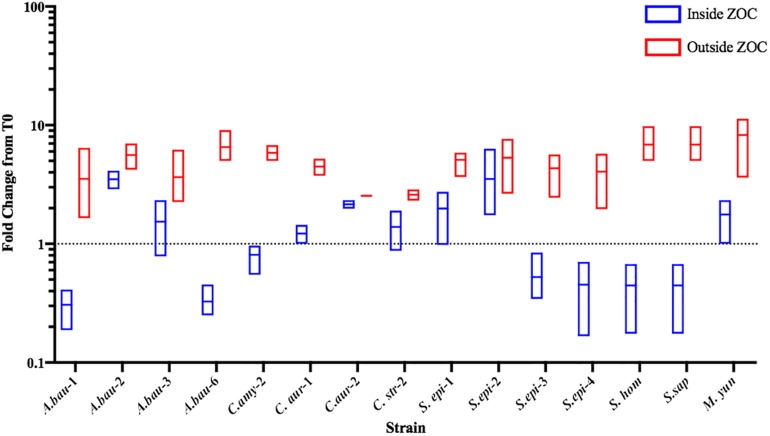
Select clinical isolates mediate bacteriostatic or bactericidal activity against *S. aureus*. The number of *S. aureus* CFU seeded within the agar (T0) was compared to the number of *S. aureus* found after 48 h of incubation. *S. aureus* CFU numbers were determined from directly adjacent to the clinical isolate spot (Inside ZOC) or from outside the ZOC and the fold change from T0 was calculated. Each box represents the data from three independent biological replicates; the horizontal line is plotted at the arithmetic mean and the length of the box represents the range. A dotted line is indicated at 1, which would represent a purely bacteriostatic interaction where the numbers of *S. aureus* at T0 and T48 were unchanged. Values below the line indicate a decrease in *S. aureus* at the 48-h time point, which indicates bactericidal activity.

Commensal bacteria can compete with other bacteria using mechanisms that either inhibit bacterial growth (bacteriostatic) or directly kill (bactericidal) the competitor. To further characterize the anti-*S. aureus* activities of the strongly inhibitory clinical isolates, the number of *S. aureus* CFU were determined from within the ZOC, directly adjacent to the clinical isolate spot (Inside ZOC), and outside of the ZOC, on the edge of the petri dish (Outside ZOC), after 48 h (T48) of incubation. These numbers were then compared to the number of *S. aureus* CFU seeded within a comparable area of the agar plate at the initiation of the experiment (T0). Of the 17 strongly inhibitory isolates, 15 developed a ZOC that was large enough (greater than 2 mm) to take accurate agar punches that fell fully within the ZOC. Of these 15 isolates, 7 mediated bactericidal activity against *S. aureus*. Most of these isolates belonged to the *Staphylococcus* genus (4/7), followed by *A. baumannii* (2/7), and *Corynebacterium* (1/7, Figure 5). Combined with the contact dependence assays, a total of 3 clinical isolates (*C. amy*-2, *S. sap*, and *S. epi*-3) produced anti-*S. aureus* activity that was independent of direct contact and was also bactericidal. This strongly suggests that these isolates directly kill *S. aureus* via the secretion of toxic compound(s).

### Basic Mechanistic Characterization of Contact-Independent Bactericidal Activity

**FIGURE 6:**
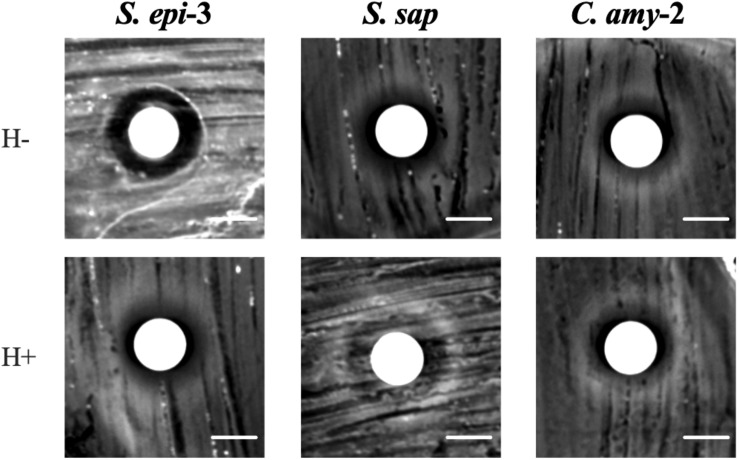
Anti-*S. aureus* activity is retained in Conditioned Cell Free Medium. *S. aureus* strains LAC or Mu50 were spread on the BHI agar surface and a sterile disk was placed in the center of the plate. Fifty μl of concentrated CCFM prepared from *C. amy*-2; (incubated with Mu50), *S. sap* (incubated with LAC), and *S. epi*-3 (incubated with LAC) that was Heat-Treated (H+) or maintained at room temperature (H–) prior to use was inoculated onto the disk and allowed to dry. Images of the ZOC were taken after 72 h of incubation. Images are representative of three independent biological replicates. Scale bar = 10 mm.

We hypothesized that clinical isolates that produced contact-independent bactericidal anti-*S. aureus* activity would do so via a secreted compound(s) that would be present in culture supernatants. To test this hypothesis, *C. amy*-2, *S. sap*, and *S. epi*-3 were independently cultured in BHIT broth, and sterile conditioned cell free medium (CCFM) was prepared. Unconcentrated CCFM and 50X concentrated CCFM were then tested in a disk diffusion assay (Figure 6 and data not shown) against the *S. aureus* strain for which they showed the most robust bactericidal activity (*C. amy*-2/Mu50, *S. sap*/LAC, and *S. epi*-3/LAC). Each of the 50X concentrated CCFM samples produced a ZOC against the tested *S. aureus* strain (Figure 6). In addition, unconcentrated CCFM derived from *S. sap* and *S. epi*-3 produced a small ZOC against *S. aureus* LAC (data not shown). To determine the thermostability of the compound(s) found in the concentrated CCFM, aliquots of CCFM were also subjected to heat treatment prior to testing for anti*-S. aureus* activity. In all cases anti-*S. aureus* activity was maintained after heat treatment (Figure 6).

**FIGURE 7:**
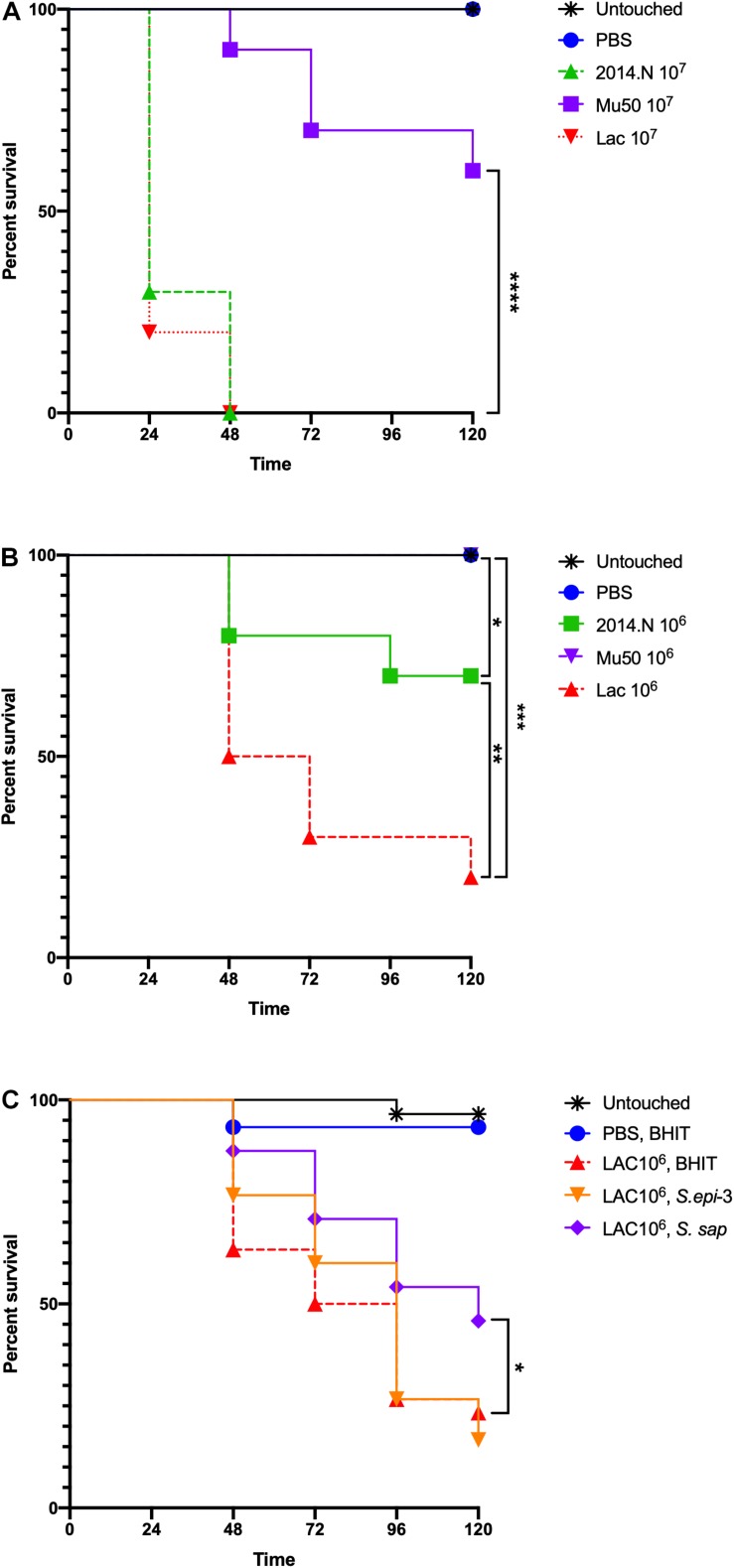
*Staphylococcus aureus*-infected *Galleria mellonella*. *Galleria mellonella* caterpillars were divided into the indicated groups and were monitored for survival over 120 h at 37°C after the indicated inoculations. **(A,B)** Untouched and PBS injected *Galleria* served as negative controls and the indicated doses of 2014.N, LAC and Mu50 were tested. **(C)** For the treatment experiments, untouched caterpillars were maintained. Additionally, PBS injected caterpillars were treated 1-h post *S. aureus* infection with CCFM prepared from BHIT in which no bacteria had been cultured to ensure no effect of dual injection on survival. Caterpillars that were injected with 10^6^ CFU of *S. aureus* strain LAC were treated 1-h post infection with BHIT CCFM (sham treatment) or *S. epi*-3 or *S. sap* derived CCFM. Kaplan-Meier survival curves were compared (excluding negative controls) using the Mantel–Cox test with Holm’s correction for multiple comparisons. In **(A,B)**, the three *S. aureus* strains were compared to identify statistically significant differences in Galleria mortality; untouched and PBS dosed caterpillars were not included in the comparisons. In **(A)**, Mu50 was significantly different than both 2014.N and LAC. In **(B)**, significant differences between the various groups are indicated. In **(C)**, BHIT treated caterpillars were compared to CCFM treated (*S. epi*-3 or *S. sap*) Galleria to identify difference; only *S. sap* was significantly different. Asterisks signifying the *P* value as follows: ^∗^*P* < 0.05; ^∗∗^*P* < 0.01; ^∗∗∗^*P* < 0.001; ^∗∗∗∗^*P* < 0.0001.

To examine the therapeutic potential of the compound(s) found within the CCFM, we next examined the ability of CCFM to rescue *S. aureus*-infected *Galleria mellonella* caterpillars. *G. mellonella* have been established as a simple infection model for several pathogens, including *S. aureus* (Desbois and Coote, 2011; Tsai et al., 2016), and have also been used to test the efficacy of antimicrobials (Desbois and Coote, 2011). Despite the usefulness of this model, little is understood about the relative virulence of different *S. aureus* strains in *G. mellonella*. We previously found that *in vitro* gene expression of important virulence factors broadly varied amongst *S. aureus* strains 2014.N, LAC, and Mu50; 2014.N expresses the highest levels followed by LAC and then Mu50 (Hardy et al., 2019). Thus, we first tested the ability of these various strains to induce *G. mellonella* mortality at various doses. The overall virulence in this model revealed that LAC induced the highest level of death, followed by 2014.N and Mu50. Indeed, infection with LAC or 2014.N killed significantly more *G. mellonella* than Mu50 at the tested doses (Figures 7A,B). These data support the notion that though *in vitro* defined virulence factor expression profiles may be helpful, they do not always directly correlate with virulence in every *in vivo* model.

To examine the therapeutic potential of the compound(s) found within the CCFM, we next tested the ability of CCFM to rescue *S. aureus*-infected *G. mellonella*. As we found Mu50 to be essentially avirulent in this model (Figures 7A,B), we focused our efforts on CCFM derived from *S. sap* and *S. epi*-3, which was most active against *S*. *aureus* LAC (Figure 2A). Treatment with *S. sap* CCFM, but not *S. epi*-3 CCFM, 1-h post infection with 10^6^ *S. aureus* LAC significantly reduced mortality of infected *G. mellonella* compared to sham treated controls (Figure 7C). Taken together, our results indicate that anti-*S. aureus* activity mediated by the various bacterial species is diverse and suggest that secreted compound(s) derived from *S. saprophyticus* may have possible future therapeutic value.

## Discussion

Humans serve as an incredibly complicated and dynamic environmental niche for microorganisms. Our understanding of this fact has been greatly enhanced by the Human Microbiome Project, which has revealed that most anatomical locations are colonized with dozens, if not hundreds of bacterial species that must compete with each other for limited nutrients (Turnbaugh et al., 2007). While large microbiota-based epidemiological studies have identified the presence of these microbes, they often fail to elucidate the molecular interactions that occur between the resident flora and how these interactions may impact incoming pathogens. In addition, interactions with opportunistic pathogens are difficult to study in particular as the factors that promote commensalism vs. pathogenesis are often ill-defined. This is especially true for *S. aureus*, which asymptotically colonizes one-quarter of the population at any given time (Kluytmans and Wertheim, 2005; Wertheim et al., 2005), while simultaneously maintaining the ability to cause severe disease. It is well-established that the composition of the host microbiota heavily influences *S. aureus* carriage (Burian et al., 2017). This is particularly true in the nasal cavity, which serves as a primary reservoir for *S. aureus* colonization (Sakr et al., 2018). As such, many microbiota studies have focused on *S. aureus* interactions with the nasal flora. However, little is known about how *S. aureus* may interact with bacteria commonly found at other anatomical sites. Thus, in a proof of concept study we set out to characterize the basic interactions of *S. aureus* with bacterial isolates obtained from various sites (wound, blood, urine, and the nasal cavity) from patients at the WRNMMC. By taking a reductionist approach, we found that the majority of clinical isolates we screened displayed some form of *in vitro* anti-*S. aureus* activity.

*En masse*, *in vitro* bacterial interaction assays against three phenotypically different *S. aureus* strains revealed that the majority of tested clinical isolates were able to inhibit *S. aureus* to some degree (Figure 1, 2). Most of the inhibitory isolates were members of the *Corynebacterium* genera (10/28), which supports well-established findings that show that the *Corynebacterium* genus heavily impacts *S. aureus* colonization and viability (Yan et al., 2013; Hardy et al., 2019). For example, we previously showed that *C. pseudodiphtheriticum*, an important community determinant of *S. aureus* nasal colonization, mediates potent strain-specific bactericidal activity against *S. aureus* via production of a secreted factor(s) (Hardy et al., 2019). The results described herein indicate that related *Corynebacterium* species (*C. aurimucosum*, *C. amycolatum*, *C. striatum*, *C. jeikeium*, and *C. tuberculostearicum*) also possess some level of anti-*S. aureus activity*. Despite this finding, it is not possible to generalize that all *Corynebacterium* species negatively impact *S. aureus*. For example, *C. accolens* has been shown to actually promote *S. aureus* nasal colonization by reducing competition from other opportunistic pathogens (Yan et al., 2013; Bomar et al., 2016). In our screen, *C. accolens* possessed no anti-*S. aureus* activity (Figure 1A). In addition, recent work from Stubbendieck et al. (2019) showed that some *Corynebacterium* species can inhibit CoNS growth through the production of siderophores that enable these species to out-compete the CoNS for available iron, and thusly influence *S. aureus* viability. Therefore, individual *Corynebacterium* species appear to have evolved independent mechanisms that allow them to either cooperate or compete with *S. aureus*. Overall, our results combined with the growing body of literature suggest that the relationships observed in microbiota-based studies can be translated into *in vitro* phenotypes, and that the *Corynebacterium* genus in particular greatly impacts *S. aureus* viability and thusly colonization.

Culture independent-identification methods have revealed that wound infections, rather than being caused by a single species, are often polymicrobial in nature (Bowler et al., 2001; Peters et al., 2012; Tay et al., 2016). Moreover, microbiota-based studies have shown that wounds that are infected with multiple bacterial species tend to have worse outcomes as compared to wounds that are infected with a single species (Dalton et al., 2011; Pastar et al., 2013). It is worth noting that bacteria within wounds have to compete for resources and must contend with the host’s immune system. To aid these processes, bacteria that commonly infect wounds have evolved multiple mechanisms that help in these responses. For example, *Pseudomonas aeruginosa* and *S. aureus* are often co-isolated from wounds (Giacometti et al., 2000; Dowd et al., 2008). *P. aeruginosa* has been found to limit *S. aureus* growth by sensing the presence of *S. aureus* peptidoglycan (Korgaonkar et al., 2013; Pastar et al., 2013). *P. aeruginosa* responds by producing pyocyanin and elastase; both of these molecules have anti-*S. aureus* properties (Korgaonkar et al., 2013). Similarly, *S. aureus* and *A. baumannii* are also commonly co-isolated from wounds. However, to our knowledge there are no published reports of cooperative or competitive interactions between these two species. Thus, we were surprised that our initial screen revealed that *A. baumannii* was the most frequently isolated species possessing anti-*S. aureus* activity (7/28, Figures 2, 3). Moreover, the various *A. baumannii* isolates displayed a wide range of anti-*S. aureus* activities that were dependent upon both the *A. baumannii* and *S. aureus* strains. Future studies that seek to understand these interactions at a molecular level will be of great interest.

In thinking about the types of inhibition that we observed, contact-dependent inhibition can be mediated by variety of different mechanisms. For example, Type VI Secretion Systems (T6SS), which are found in many Gram-negative species, require physical contact and involve injection of toxic compounds directly into competitor cells (Coulthurst, 2019). Similarly, though mechanistically divergent from the T6SS, the Esx secretion pathway, which is broadly distributed amongst Gram-positive bacteria, also requires physical contact between competing bacterial species to mediate growth inhibition via toxic compounds (Whitney et al., 2017). In both these examples, only target cells that are physically touching the inhibitory cells are negatively impacted. In contrast contact-independent growth inhibition is typically mediated by toxic compounds that are synthesized and then secreted by the inhibitory species as a means to kill/prevent the growth of a competitor; no cell-to-cell contact between the two species is required. This approach is a common mechanism that is used by various microbes across multiple ecological niches and these compounds can include bacteriocins, secondary metabolites, and other small molecules (Zipperer et al., 2016; Terra et al., 2018). Finally, it is worth noting that some antagonistic interactions are more complex and can involve both contact-independent and dependent mechanisms. For example, initial physical contact between *Streptococcus pneumoniae* and *S. aureus* induces *S. pneumoniae* to generate and secrete hydrogen peroxide that can then kill *S. aureus* (Khan et al., 2016; Wu et al., 2019). Similarly, *P. aeruginosa* can physically senses *S. aureus*, which leads to global changes in transcription, resulting in the secretion of multiple compounds that have anti *S. aureus* activity (Korgaonkar et al., 2013; Filkins et al., 2015). Thus, of the 10 strongly inhibitory clinical isolates that produced a ZOC against *S. aureus* that was dependent on direct contact (Figure 4), some of these may require initial physical contact with *S. aureus* as a way to stimulate production of a toxic compound(s) or a secondary metabolite into the surrounding agar that can alter the pH or other environmental conditions in such a way as to impact *S. aureus* viability in that region. Undoubtedly, various species and strains utilize a diverse number of mechanisms to inhibit *S. aureus*.

Recently, there has been a renewed interest in the use of bacterial-derived compounds as novel therapeutics to treat highly drug resistant infections. Indeed, these compounds are potentially even more valuable because of the dearth of new antibiotics that are entering the market for human use. Our studies identified several isolates that inhibited *S. aureus* growth independent of physical contact, presumably through the activity of a secreted and diffusible compound(s) (Figures 1, 4). These isolates exclusively belonged to the *Corynebacterium* and *Staphylococcus* genera. Given that *Corynebacterium* and *Staphylococcus* are the primary genera that have been found to inhibit *S. aureus* growth on the skin and within the nasal cavity, it is clear that there appears to be a selective pressure for members of these genera to compete with *S. aureus*. It is worth noting that a portion of the identified isolates mediated killing activity (Figure 5); *S. epidermidis* represented the majority of the isolates that mediated bactericidal activity. This finding is likely not unexpected given that *S. epidermidis*, a common member of the human microbiota, has been found to actively compete with *S. aureus* by a variety of mechanisms: production of *S. aureus*-specific anti-microbial peptides, production of anti-biofilm compounds, and rapid and efficient nutrient acquisition (Lina et al., 2003; Iwase et al., 2010; Nakatsuji et al., 2017).

While the finding that *S. epidermidis* inhibits *S. aureus* is not surprising, to our knowledge our results are the first to show that *Staphylococcus saprophyticus* has anti-*S. aureus* activity (Figures 4–6). Moreover, we observed that treatment with *S. saprophyticus* CCFM was able to rescue survival of *S. aureus*-infected *G. mellonella* caterpillars (Figure 7C). *S. saprophyticus* is the second most common cause of bacterial urinary tract infections (UTIs) and is not associated with the healthy urinary tract (Marrie et al., 1982). Thus, *S. saprophyticus* presumably must out-compete normal urinary tract flora during the process of colonization and ultimate disease causation. Given that *S. aureus* can also infrequently colonize the urinary tract and cause UTIs, it’s interesting to speculate that *S. saprophyticus* has evolved to kill *S. aureus* as a means to prevent competition for this niche.

We note that secreted bactericidal compound(s) from some of the characterized isolates may have the potential to be developed for use as novel therapeutics to treat or prevent *S. aureus*-mediated infection. This ascertain is supported by the fact that anti-*S. aureus* activity was retained in CCFM from the three tested isolates (Figure 6), suggesting that these species negatively impact *S. aureus* viability most likely through the secretion of a toxic compound(s). Thought the nature of these compound(s) are unclear, they may include compounds like lantibiotics (McAuliffe et al., 2001), which are peptide antibiotics that are produced by a broad range of Gram-positive bacteria, including *Staphylococcus*. Genes that code for lantibiotics are often located on plasmids and other mobile genetic elements, and have a wide range of target-species specificity. Lantibiotics from closely related Staphylococcal species, such as epidermin (Götz et al., 2014), have been found to have potent inhibitory activity against *S. aureus*, including MRSA. It is possible that the anti-*S. aureus* activity we observed from *S. saprophyticus*, and the other Staphylococcal tested species, is the result of a lantibiotic that maintains potent inhibitory properties. Combined, our results indicate that many Staphylococcal species have evolved strategies to compete with *S. aureus*.

While this work was designed as a proof of concept study to explore the extent of anti-*S. aureus* activity exhibited by various microbes, we acknowledge that there are limitations to the study. For example, while the patient population at WRMMC is fairly diverse, given that many of the patients are soldiers that may have incurred traumatic injuries during the course of their service, a substantial proportion of isolates were obtained from wounds; this undoubtedly affected the types of species of bacteria that we ultimately screened. In addition, while this study described the basic molecular mechanisms of these interactions, a more detailed study will be required to clearly identify specific compounds and/or mechanisms of action that are responsible for anti- *S. aureus* activity.

In summary, this proof of concept study indicates that multiple bacterial species possess strain-specific anti-*S. aureus* activity when co-cultured in a bacterial interaction assay. This study further highlights the multifarious nature of polymicrobial interactions, which remain poorly understood. Furthermore, this work expands upon the growing body of literature that supports that the study of ‘bacterial warfare’ and the toxic compounds created by microbes as a means to compete with one another may be a ‘next best option’ for the identification of novel therapeutics that will help in overcoming the significant increase in antimicrobial resistance that threatens the health and wellbeing of the population (Zipperer et al., 2016; Nakatsuji et al., 2017; Stubbendieck et al., 2019). As such, we hypothesize that several of the inhibitory isolates identified in this study may produce toxic compounds that have the potential to be used as novel therapeutics or intervention strategies. Our future work will pursue elucidation of the molecular mechanism by which both *A. baumannii* and *S. saprophyticus* inhibit *S. aureus*. Overall, our findings support the continued study of polymicrobial interactions as a means to identify novel therapeutics and/or molecular targets of *S. aureus* and other pathogens.

## Data Availability Statement

The datasets generated for this study can be found in the NCBI, GenBank, MN175920–MN175947.

## Author Contributions

BH and DM designed the research study. EK and JB provided the clinical bacterial isolates utilized in all experiments. BH, GB, KH, AA, and SS performed the experiments. BH, GB, and DM analyzed the data. BH wrote the manuscript. All authors contributed substantially to revisions and approved the final manuscript.

## Conflict of Interest

The authors declare that the research was conducted in the absence of any commercial or financial relationships that could be construed as a potential conflict of interest.

## Footnotes

1 http://rdp.cme.msu.edu

2 https://blast.ncbi.nlm.nih.gov
